# The Predominance of Tobacco Propensities and Tobacco-Related Oral Lesions in Textile Mill Workers of Bhopal: A Cross-Sectional Study

**DOI:** 10.7759/cureus.41085

**Published:** 2023-06-28

**Authors:** Arshdeep Kaur, Neeraj S. Chauhan, Sahana Shivakumar

**Affiliations:** 1 Public Health Dentistry, Bhabha College of Dental Sciences, Bhopal, IND; 2 Public Health Dentistry, People's College of Dental Sciences and Research Centre, Bhopal, IND

**Keywords:** prevalence, tobacco habit, oral mucosal lesions, cross-sectional study, industrial workers

## Abstract

Introduction: Low wages, long work hours, and stressful working conditions predominantly affect the oral and general health of industrial workers, which in turn result in their tobacco consumption. This cross-sectional study examined the prevalence of tobacco use and its associated oral lesions among textile mill workers in Bhopal, India. Oral cancer and premalignant lesions are significantly increased by smoking and chewing tobacco. The study's objective was to assess and record the prevalence of oral mucosal lesions linked to tobacco use in different age groups among Bhopal textile industry workers.

Methodology: A cross-sectional study was conducted among 583 textile mill workers. Data were collected using a questionnaire and the WHO Oral Health Assessment Form 2013. IBM SPSS Statistics for Windows, Version 29 (Released 2022; IBM Corp., Armonk, New York, United States) was used for the statistical analysis. Variables were compared using the mean, percentage, and standard deviation. The chi-square test and Fisher's exact test were used to compare the distribution of gender, tobacco habits, and oral mucosal lesions in different age groups.

Results: Males made up 69.1% of the workforce. A clear male preference was noted (P ≤ 0.001). About 64.7% of the workers did not have any tobacco-related habits, 20.8% used smokeless tobacco, 7.9% used a smoking form of tobacco, and 6.7% used both. Older age groups, 31-45 and >46 years old, had a higher proportion of users of smokeless tobacco (P ≤ 0.001). The most commonly reported oral mucosal lesions were ulcerative conditions in 6.9%, followed by oral submucous fibrosis (OSMF), keratotic lesions, and leukoplakia in 5.0%, 4.1%, and 3.6% of the study population, respectively. Leukoplakia and OSMF were prevalent in the 31-45-year age group, while ulcerative lesions were more prevalent in the 18-30-year age group (P ≤ 0.001).

Conclusion: Workers at textile mills were more likely to use a smokeless form of tobacco. Older age groups had higher rates of smokeless tobacco use as compared to smoking, which was more prevalent in the younger age group. Oral mucosal lesions in older age groups frequently include OSMF and leukoplakia. The main reasons for engaging in the tobacco use habit were stress and a lack of awareness. Oral hygiene was a neglected entity among workers.

## Introduction

The Industrial Revolution of the 18th century was the key development that significantly impacted occupational health. As a result of the Industrial Revolution, work is now done in factories in urban areas [1]. The growth of industrial activities and the resulting increase in employment have raised people's standards of living and quality of life. However, offering workers safety and health at the workplace is crucial given the rapid economic expansion and industrial advancement. Therefore, a key determinant of health is the workplace environment [2].

The Indian textile sector is strong at every point along the value chain, from garments to home furnishings, from natural to synthetic fibres. It contributes 6% to the gross domestic product (GDP) of the country and 13% to exports [3]. The industry employs more people than any other sector after agriculture [3]. Nowadays, this industry is implementing innovative ideas to manufacture polyester fibre, the bold example of which is the conversion of plastic bottle waste into fabric while diligently handling the environmental pollution problem. Accordingly, our study centre is in the same industry.

One of the biggest problems currently facing global public health is socioeconomic health disparities. Community growth and capacity are influenced by individual health and, by extension, societal health [4]. Many factory workers are underpaid and frequently work long shifts. All of these factors contribute to stress, which is closely linked to tobacco use and has negative effects on workers' general and oral health with a higher risk of oral cancer and other potentially malignant illnesses (OPMDs) [5]. The oral mucosa changes could just start as a change in surface colour or texture. As a result, lesions are rarely noticed by tobacco users because of their clinical presentation's apparent innocence [6].

The world's most significant preventable public health problem is tobacco consumption, which is expected to be the leading cause of death globally. One of the countries with the highest rates of oral cancer linked to tobacco use is India [7]. With almost one-third of the population consuming tobacco, India, the second most populous nation in the world, significantly contributes to the global burden of diseases related to tobacco use. Every year, more than one million Indians die from tobacco smoking. However, because of a lack of publicly accessible tools for cessation support and a lack of social norms that encourage quitting, India does not have a widespread practice of stopping [8].

Dental teams, being in a unique position, can help patients adopt healthier choices. Smoking and tobacco consumptions are addictive behaviours with strong social associations that are very difficult to stop. However, advice, support, and encouragement from primary healthcare professionals can significantly impact those who want to quit [5]. Given that India accounts for 20% of the global burden of occupational diseases, the incidence of occupational disorders in the country is cause for concern [9]. Therefore, reliable and current data on national tobacco usage rates by job category are crucial to distribute and target preventive healthcare activities at a national level in the most effective manner [10]. All of these factors were taken into consideration as the current study sought to determine the prevalence of oral lesions linked to tobacco use among Bhopal's textile industry workers. The paucity of literature about textile mill workers in Bhopal also acted as a precursor for us to conduct this study.

## Materials and methods

A cross-sectional study was performed at a private textile mill (Badri Cotsyn and Badri EcoFibre Pvt. Ltd. in Bhopal, Madhya Pradesh, India) with two manufacturing plants situated in the Bhopal Industrial Area. The study was split into two phases: First, the examiner completed an interview-based, pre-structured, validated questionnaire that inquired about oral hygiene practices, dental appointments, tobacco use, awareness of the adverse consequences of tobacco, and dietary habits (cane sugar, sugar-sweetened beverages, rice, and dairy products were considered to have higher sugar consumption, whereas vegetables, grains, beans, nuts, and meat were considered to have lower sugar consumption).

Validity of the questionnaire

The researchers created a 10-variable questionnaire. Using Pearson's correlation coefficient, the convergent validity of the questionnaire items was assessed. Every question was regarded as legitimate because the resulting value exceeded the critical value for each and was extremely important. The degree of significance was evaluated by a two-team expert panel. Item-Content Validity Index (I-CVI) for each item received a "1" to indicate complete agreement. It was a pertinent questionnaire because its scale level-CVI (S-CVI) was based on the total of all I-CVI scores of 0.9.

Second, a clinical examination was performed based on the 2013 WHO Oral Health Assessment Form (For Adults) [11]. The investigation was conducted by a single calibrated examiner using a type III examination technique (inspection using a mouth mirror and explorer with good illumination) under rigorous infection control guidelines. The oral clinical examination included dentition status, periodontal status, dental erosion, and oral mucosal lesions. The diagnosis of oral mucosal lesions was based on clinical examination only. The study included only employees who were willing to participate, available during the study period, and employed for at least six months. The study excluded employees who were chronically unwell or on medications and those unable to collaborate or communicate. We used a program called Raosoft sample size calculator (Raosoft Inc., Seattle, Washington, USA) to calculate the sample size with a 4% margin of error, 95% confidence level, and 50% response distribution. All of these numbers were substituted, resulting in a study sample size of 583.

The Institutional Ethics Committee examined and approved the study’s design (approval no. BCDS/DEAN/2022/2473). After informing the subjects of the research goal and methodology, the relevant textile mill authorities authorised the study. The subjects then provided written informed consent.

Statistical analysis

IBM SPSS Statistics for Windows, Version 29 (Released 2022; IBM Corp., Armonk, New York, United States) was used to analyse the responses gathered. The mean, standard deviation, and proportions were calculated for each clinical measure. To compare tobacco-use behaviours and oral mucosal lesions across age groups, Fisher's exact test was performed. The level of significance was established at 5%.

## Results

The study surveyed a total of 583 employees from two manufacturing facilities at the textile mill who met the inclusion and exclusion criteria. The male population was larger (statistically significant at P ≤ 0.001), with 69.1% of the sample being males as against 30.9% females. The majority of the study participants were in the 31-45-year age group. The fewest participants were in the >46-year age group. The distribution of the study participants by age and gender is presented in Table 1.

**Table 1:** Distribution of gender among age groups *: significant; df: degrees of freedom

Age group	Males		Females		Total	
	N	%	N	%	N	%
18-30 years old	159	79.1	42	20.9	201	34.5
31-45 years old	154	70.6	64	29.4	218	37.4
>46 years old	90	54.9	74	45.1	164	28.1
Total	403	69.1	180	30.9	583	100.0
Chi-square statistics	25.212					
df	2					
P-value	<0.001*					

The examiner distributed the questionnaire to the 583 responders. Most participants used a toothbrush and toothpaste once a day for oral hygiene, and they only went to the dentist’s office in cases of extreme discomfort or urgency. Pan masala is a smokeless tobacco product that was most often used. Most of the respondents had been using smokeless tobacco for the past six to 10 years. The main factor that led to the development of the tobacco use habit was stress. The majority of the participants were aware that tobacco consumption is harmful to them, but few of them recognized that it may cause oral precancerous lesions, which can eventually progress to oral cancer. The frequency distribution of the study variables is shown in Table 2.

**Table 2:** Descriptive analysis: frequency distribution of the study variables

Variable	Category N (% percentage)							
Diet								
Sugar consumption	Higher				Lower			
	133 (22.8)				450 (77.2)			
Oral hygiene practices								
Method	Toothbrush		Finger				Others	
	395 (67.7)		186 (31.96)				2 (0.34)	
Material	Toothpaste		Powder				Others	
	462 (79.2)		119 (20.46)				2 (0.34)	
Frequency	Twice		Once				< Once daily	
	53 (9.1)		530 (90.9)				0 (0.0)	
Abnormal oral habits								
Smoking	Present		Absent				Ex-smoker	
	85 (14.6)		493 (84.5)				5 (0.9)	
Type	Cigarette		Bidi				Others	
	55 (64.7)		30 (35.3)				0 (0.0)	
Smokeless tobacco	Present		Absent				Ex- chewer	
	160 (27.5)		383 (65.6)				40 (6.9)	
Type	Pan masala		Khaini				Gutka	
	103 (64.4)		57 (35.6)				0 (0.0)	
Frequency of smoking	1-5 years	6-10 years		11-20 years		21-40 years		>40 years
	53 (62.4)	18 (21.1)		14 (16.5)		0 (0.0)		0 (0.0)
Frequency of chewing	1-5 years	6-10 years		11-20 years		21-40 years		>40 years
	42 (26.3)	101 (63.1)		17 (10.6)		0 (0.0)		0 (0.0)
Reasons for starting the habit	Peer pressure	Curiosity		Role model		Stress		Low self-esteem
	32 (13.1)	32 (13.1)		2 (0.8)		179 (73.0)		0 (0.0)
Awareness								
Harmful effects of tobacco	Present				Absent			
	347 (59.5)				236 (40.5)			
Oral precancerous lesions	Present				Absent			
	117 (20.1)				466 (79.9)			
Oral cancer	Present				Absent			
	148 (25.4)				435 (74.6)			
Willingness to stop tobacco	Present				Absent			
	180 (30.9)				403 (69.1)			
Ever tried to quit the addiction	Present				Absent			
	140 (24.0)				443 (76.0)			
Any counseling before	Yes				No			
	30 (5.1)				553 (94.9)			

The overall decayed, missing, and filled teeth (DMFT) scores showed an increase with increasing age, from 2.37 ± 0.33 in the 18-30-year age group to 5.87 ± 0.82 in the >46-year age group, which was statistically significant at P ≤ 0.001. Pockets tended to be greater in the older age groups. Loss of attachment of 4-5 mm was noted in 13.8%, 6-8 mm in 6.6%, and 9-11 mm in 3.1% of the textile factory workers; notably higher percentages of loss of attachment were seen in the older age groups (demonstrating statistical significance at P ≤ 0.001). Erosive lesions were noted in 20.8% of the study population in the enamel layer, 14.1% in the dentine, and 3.6% in the pulp. Erosive lesions were noted on the higher end of the study population owing to the chemicals used in the textile industry. Table 3 displays the results of the study population’s dentition status, periodontal status, and erosive lesions.

**Table 3:** Oral clinical examination showing the dentition status, periodontal status (CPI Modified and loss of attachment), and dental erosion in textile mill workers DMFT: decayed, missing, and filled teeth; ANOVA: analysis of variance; SD: standard deviation; CPI: community periodontal index; *: significant

Variable	Category	Age groups in years				Statistical test used	P-value
		18-30 N (%)	31-45 N (%)	>46 N (%)	Total N (%)		
Dentition status (Mean ± SD)	DMFT	2.37 ± 0.33	3.70 ± 1.04	5.87 ± 0.82	4.1 ± 1.5	519.702 (ANOVA statistics)	≤0.001*
CPI Modified	Gingival bleeding	28 (13.9%)	24 (11.1%)	11 (6.7%)	63 (10.8%)	47.399 (Chi-square statistics)	≤0.001*
	Pocket 4-5 mm	22 (10.9%)	36 (16.5%)	41 (25.1%)	99 (16.9%)		
	Pocket 6 mm or more	9 (4.5%)	17 (7.8%)	22 (13.4%)	48 (8.3%)		
Loss of attachment	0-3 mm	177 (88.0%)	158 (72.5%)	111 (67.6%)	446 (76.5%)	25.402 (Chi-square statistics)	≤0.001*
	4-5 mm	13 (6.5%)	34 (15.6%)	33 (20.2%)	80 (13.8%)		
	6-8 mm	9 (4.5%)	17 (7.8%)	13 (7.9%)	39 (6.6%)		
	9-11 mm	2 (0.9%)	9 (4.1%)	7 (4.3%)	18 (3.1%)		
Dental erosion	No erosion	120 (59.7%)	169 (77.5%)	70 (42.7%)	359 (61.6%)	210.986 (Fisher’s exact test statistics)	≤0.001*
	In the enamel	44 (21.9%)	4 (1.8%)	73 (44.5%)	121 (20.8%)		
	In the dentine	37 (18.4%)	45 (20.6%)	0 (0.0%)	82 (14.1%)		
	In the pulp	0 (0.0%)	0 (0.0%)	21 (12.8%)	21 (3.6%)		

About 64.7% of workers did not have any tobacco-related habit, 20.8% used smokeless tobacco, 7.9% used a smoking form of tobacco, and 6.7% used both. While the older age groups, 31-45 and >46 years old, had a higher proportion of users of smokeless tobacco (24.8% and 29.9%, respectively, demonstrating statistical significance at P ≤ 0.001), smoking was more prevalent in the 18-30-year age group (17.4%). Table 4 displays the study population’s tobacco use by age group.

**Table 4:** Distribution of tobacco habits in the study population based on age groups * = Significant, df = degrees of freedom

Age group	No habits		Smoking		Smokeless tobacco		Both smoking and smokeless tobacco		Total	
	N	%	N	%	N	%	N	%	N	%
18-30 years old	126	62.7	35	17.4	18	9.0	22	10.9	201	34.5
31-45 years old	147	67.4	4	1.8	54	24.8	13	6.0	218	37.4
>46 years old	104	63.4	7	4.3	49	29.9	4	2.4	164	28.1
Total	377	64.7	46	7.9	121	20.8	39	6.7	583	100
Fisher's exact test statistics	68.631									
df	6									
P-value	< 0.001*									

**Figure 1:**
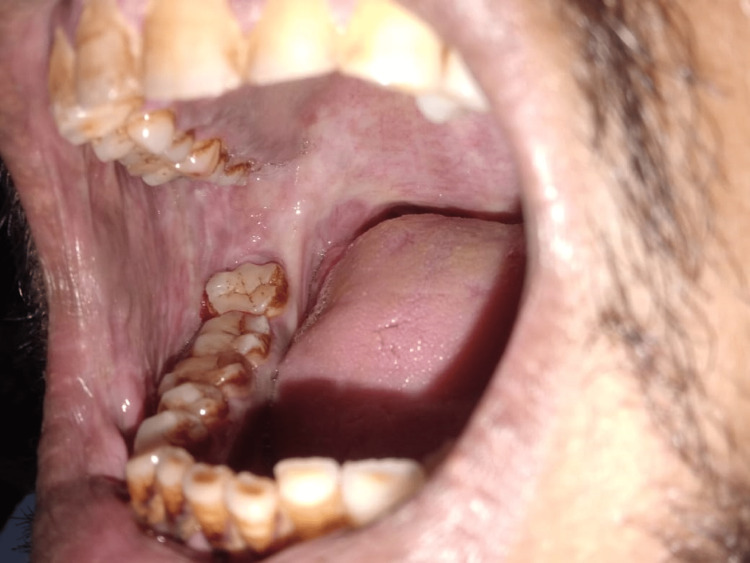
Oral submucous fibrosis, right buccal mucosa The buccal mucosa shows a loss of elasticity and fibrous banding.

**Figure 2:**
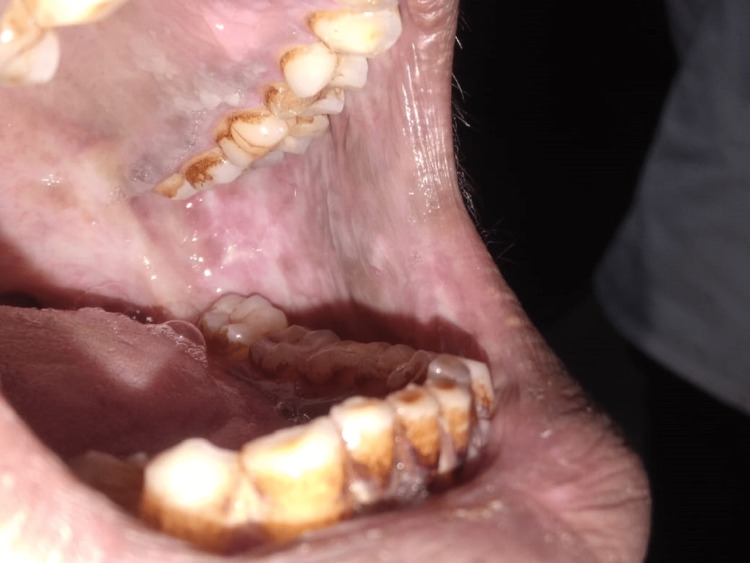
Oral submucous fibrosis, left buccal mucosa Tooth staining: These extrinsic stains are due to long-term tobacco use.

**Figure 3:**
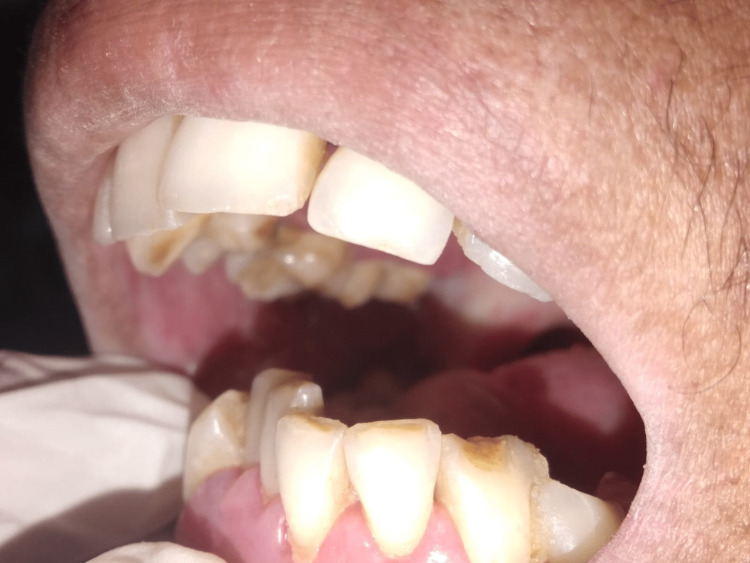
Restricted mouth opening due to OSMF OSMF: oral submucous fibrosis

**Figure 4:**
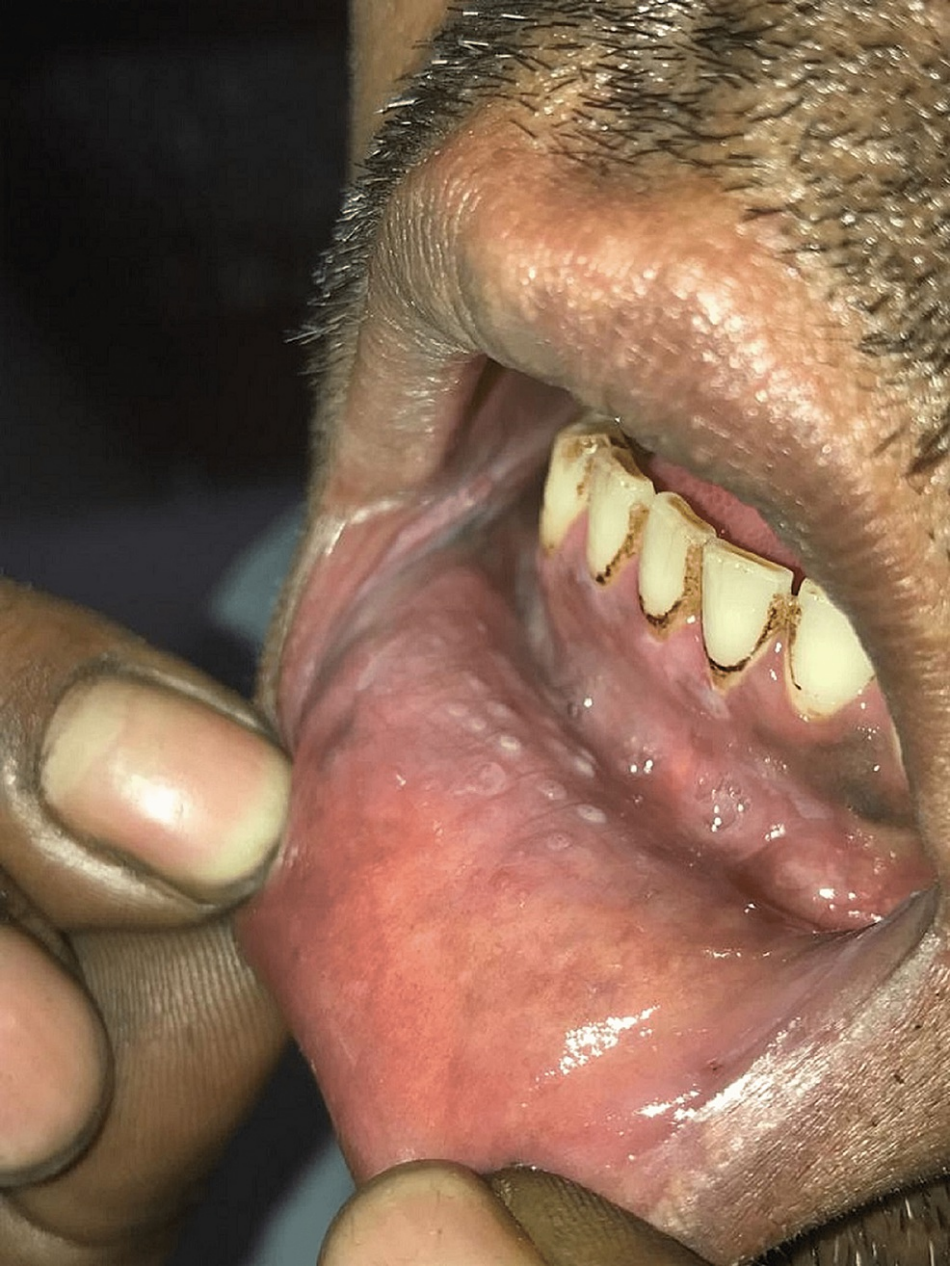
Patient's lower labial sulcus displaying tobacco pouch keratosis in areas where quid was kept for long periods

The buccal mucosa was the site of the majority of leukoplakia and OSMF lesions (81.0% and 65.5%, respectively, demonstrating statistical significance at P ≤ 0.001), whereas the labial mucosa was the site of the majority of tobacco-related aphthous ulcers (57.5%). The vestibular areas, which correlate to the area where the tobacco is kept in the mouth, commonly had keratotic lesions (statistically significant at P ≤ 0.001), as shown in Table 5. The buccal mucosa, labial mucosa, vestibule, and corners of the mouth were the anatomic regions in the oral cavity where tobacco-related oral lesions were most prevalent (Figures 1, 2, 3, 4).

**Table 5:** Distribution of oral lesions according to the location in the oral cavity *: significant; df: degrees of freedom; OSMF: oral submucous fibrosis

Condition	Location									
	Buccal mucosa		Commissures		Labial mucosa		Vestibular regions		Total	
	N	%	N	%	N	%	N	%	N	%
Aphthous ulcers	11	27.5	0	0.0	23	57.5	6	15.0	40	35.1
Leukoplakia	17	81.0	0	0.0	4	19.0	0	0.0	21	18.4
OSMF	19	65.5	10	34.5	0	0.0	0	0.0	29	25.4
Keratotic lesions	1	4.2	0	0.0	1	4.2	22	91.7	24	21.1
Total	48	42.1	10	8.7	28	24.6	28	24.6	114	100.0
Fisher’s exact test statistics	118.553									
df	9									
P-value	≤0.001*									

**Figure 5:**
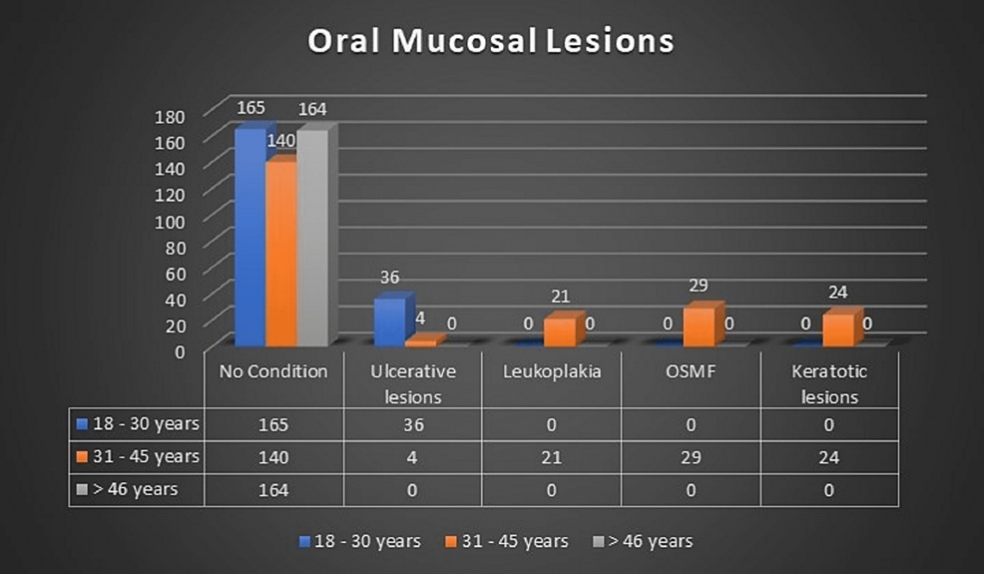
Distribution of tobacco-related oral lesions based on age group

Table 6 and Figure 5 show the distribution of tobacco-related oral lesions (OPMDs) by age. The most often reported oral mucosal lesions were aphthous ulcers, which affected 6.9% of the research population. Oral submucous fibrosis, keratosis, and leukoplakia affected 5.0%, 4.1%, and 3.6% of the study population, respectively. Some of the participants (80.4%) had no oral mucosal problems to report. Comparing the occurrence of oral lesions by age group, leukoplakia and OSMF were only found in the 31-45-year age group, while ulcerative lesions were more prevalent in the 18-30-year age group (statistically significant at P ≤ 0.001).

**Table 6:** Oral mucosal lesions distribution among the study population based on the age groups *: significant, df: degrees of freedom; OSMF: oral submucous fibrosis

Age group	No condition		Aphthous ulcers			Leukoplakia		OSMF		Keratotic lesions		Total		
	N	%	N	%	N		%	N	%	N	%	N	%	
18-30 years old	165	82.1	36	17.9	0		0.0	0	0.0	0	0.0	201	34.5	
31-45 years old	140	64.2	4	1.8	21		9.6	29	13.3	24	11.0	218	37.4	
>46 years old	164	100.0	0	0.0	0		0.0	0	0.0	0	0.0	164	28.1	
Total	469	80.4	40	6.9	21		3.6	29	5.0	24	4.1	583	100.0	
Fisher's exact test statistics	194.269													
df	8													
P-value	<0.001*													

## Discussion

The amount of industrial growth reflects the expansion of a nation since employees in this sector make up a significant portion of the total population and live in a highly complicated environment; therefore, the health of this group reflects the health of the community. The lack of medical and dental facilities on the factory grounds may be the reason for the poor oral health of textile mill workers [12].

It has been suggested that personal risk factors, including a bad lifestyle and unfavourable psychosocial circumstances, play a significant role in the aetiology of addiction to tobacco-related behaviours. The employees' habits of chewing tobacco, drinking, and smoking increase their risk of oral disorders [13]. The study showed a definite preference for men. Most participants were between 31 and 45 years old. The propensity of younger age groups to investigate alternative income sources may be one explanation for this phenomenon.

According to the statistics from the Madhya Pradesh state’s data from the Global Adult Tobacco Survey (GATS) India (2016-2017) [14], the prevalence of tobacco smoking was 10.2%, and smokeless tobacco users represented 28.1% of tobacco users. In our study, smokers made up 7.9% of the population, while smokeless tobacco users made up 20.8% of users. The data are comparable in both studies.

According to our research, smoking was more common among those between the ages of 18 and 30 years old, whereas smokeless tobacco use was more widespread among the older age groups. Our findings were at odds with a study by Chaudhary et al. [15], which shows that most smokers (27.1%) were under the age of 55 years old, whereas tobacco chewers (29.2%) were between the ages of 25 and 34 years old. Both studies found that tobacco chewing and smoking were prevalent addictions and that mouth ulcers and trismus were typical symptoms.

In our study, among the oral mucosal lesions, aphthous ulcers were the most frequently reported condition at 6.9%, followed by leukoplakia at 3.6%. The majority of ulcerative lesions were seen in people between the ages of 18 and 30 years old, while leukoplakia was only found in people between the ages of 31 and 45 years old. A clinical investigation on oral mucosal lesions in patients visiting a tertiary care institution in central India conducted by Goyal et al. [16] found that aphthous ulcers were the most frequent mucosal lesion (44.5%), followed by leukoplakia. Leukoplakia was found in the senior age group (61-80 years old), whereas most aphthous ulcer patients (54.7%) were in the younger age group (21-40 years old). The incidence of ulcerative lesions was higher in the younger age group and the prevalence of leukoplakia rose with age in both trials, so the age distribution of these lesions was comparable.

The lower cost and difficulty in recognizing a person’s habit, which leaves family members unaware of loved ones’ use, may contribute to the higher prevalence of smokeless tobacco users. Another explanation for India’s high tobacco usage rates is their customs, which allow people to ingest tobacco and areca nuts in various ways [5].

To enhance working and social conditions, restrictions are required at both the public and private (mill owners) levels. There must be educational materials about oral health, the dangers of smoking, and the advantages of quitting displayed on mill properties. For the industrial sector specifically, routine dental care services should be offered to detect early indications and arrange preventive measures. Public health professionals have a responsibility to champion change to advance oral health and lessen inequality. On the underlying social determinants of health, coordinated and integrated action is required, with an emphasis on enhancing the living, working, and social conditions of the most vulnerable people.

The cross-sectional methodology and the potential for selection bias were the study's weaknesses. However, selection bias would more likely result in an underestimation of the issue, given that textile mills with the worst conditions would not have been readily available for (voluntary) inspection.

## Conclusions

Compared to smoking, Bhopal's textile mill workers used smokeless tobacco more frequently. The age group of 31 to 45 years old, comprised of employees who had been consuming tobacco for the past six to 10 years, had the highest prevalence of OSMF and leukoplakia. The buccal mucosa was the region where OSMF and leukoplakia were most frequently seen. In some instances, fibrotic bands of OSMF reached the mouth's corner. With advancing age, periodontal diseases were becoming worse. Stress and a lack of knowledge about how tobacco use might result in precancerous diseases and oral cancer were the main factors that contributed to the tobacco use habit. Workers at textile mills generally disregarded their oral hygiene needs.

## Appendices

QUESTIONNAIRE

The Predominance of Tobacco Propensities and Tobacco-Related Oral Lesions in Textile Mill Workers of Bhopal: A Cross-Sectional Study

Principal investigator: Dr. Arshdeep Kaur                                      

Demographics Data:                                                   Date:

1.       PATIENT'S NAME:  Mr./Ms.

2.       AGE:

3.       GENDER:

4.       INCOME:

5.       DURATION OF EMPLOYMENT:

 1. Up to 2 years         2. From 2 to 5 years      3. From 6 to 10 years      4. From 11 to 15 years                                      

6.       DIET HISTORY

a)      Vegetarian / mixed

b)      Sugar consumption            Higher   (     )             Lower   (     ) 

c)      Diet diary   

**Table 7:** Diet diary (24 hours before)

FORM	CONSISTENCY	FREQUENCY	TIME
SOLID	STICKY	ONCE A DAY	WITH MEALS
LIQUID	NON STICKY	TWICE A DAY	IN BETWEEN MEALS
BOTH	BOTH	3 TIMES PER DAY	BOTH

7.       SYSTEMIC DISEASES:    YES/NO

          IF YES, TREATMENT:     YES/NO

**Table 8:** Systemic diseases

DIABETES	HYPERTENSION
CHRONIC HEART DISEASES	ANY OTHER

8.        ORAL HYGIENE PRACTICES

**Table 9:** Oral hygiene practices

METHOD	MATERIAL	FREQUENCY	TIME
TOOTH BRUSH	TOOTH PASTE	TWICE OR MORE DAILY	MORNING
FINGER	TOOTH POWDER	ONCE DAILY	NIGHT
OTHERS	OTHER	< ONCE DAILY	BOTH

9.        VISIT TO DENTIST:  YES/NO

REASONS IF YES      1. PAIN   2. FILLING   3. EXTRACTION   4. SCALING   5. CHECK-UP   6. COMBINATION     7. OTHERS

10.       ABNORMAL ORAL HABITS                                                                                                                  

a.    SMOKING                  PRESENT     (     )            ABSENT     (     )          EX-SMOKER     (     )   

       TYPE                          CIGARETTE  (      )           BIDI     (     )        

 b.   SMOKELESS               PRESENT     (     )            ABSENT     (     )          EX-CHEWER     (     )   

       TYPE                          PAN MASALA     (     )      KHAINI       (     )          GUTKA     (     )

c.      FREQUENCY OF CHEWING                                                                                                                                                                                1. 1-5 years        2. 6-10 years        3. 11-20 years        4. 21-40 years        5.  >40 years

d.      FREQUENCY OF SMOKING                                                                                                                                                                               1. 1-5 years         2. 6-10 years        3. 11-20 years        4. 21-40 years       5.  > 40 years

e.      REASONS FOR STARTING TOBACCO                                                                                                                                                             1. PEER PRESSURE       2. CURIOSITY      3. ROLE MODEL      4. STRESS      5. LOW SELF-ESTEEM

f.      HABIT OF ALCOHOL DRINKING:      Yes/No

g.     AWARENESS

1)      AWARENESS OF HARMFUL EFFECTS OF TOBACCO                                           PRESENT/ABSENT

2)     AWARENESS OF ORAL PRECANCEROUS LESION                                                PRESENT/ABSENT                             

3)     AWARENESS OF ORAL CANCER                                                                            PRESENT/ABSENT

4)     WILLINGNESS TO STOP TOBACCO                                                                        YES/NO             

5)     EVER TRIED TO QUIT THE ADDICTION                                                                  YES/NO

6)     ANY  COUNSELLING BEFORE                                                                                 YES/NO

         IF YES (PLACE & DURATION)                            
